# Euroanalysis XVII—analytical chemistry for human well-being and sustainable development

**DOI:** 10.1007/s00216-014-7731-x

**Published:** 2014-04-02

**Authors:** Ewa Bulska

**Affiliations:** Faculty of Chemistry, University of Warsaw, Pasteura 1, 02-093 Warsaw, Poland

The first Euroanalysis conference took place in Heidelberg, Germany, more than 30 years ago. Since 1972, the meeting have focused on various analytical chemistry issues, and been established as a forum for the analytical community from academia, governance, and industry in Europe with worldwide recognition. Euroanalysis, held biannually, with the venue rotating among European countries, was hosted so far by Austria, Belgium, France, twice in Germany, Hungary, Ireland, Italy, Portugal, Serbia, Spain, Switzerland, and twice in Poland (the last in 2013). The main organizer of the Euroanalysis conferences is the Division of Analytical Chemistry of the European Association for Chemical and Molecular Sciences (EuCheMS).

The 17th European Conference on Analytical Chemistry, held in Poland from August 25 to 29, 2013, was organized by the Polish Chemical Society and Committee on Analytical Chemistry of the Polish Academy of Sciences. Supported by EuCheMS, IUPAC, and the Polish Ministry of Science and Higher Education, the event was jointly organized by the Faculties of Chemistry of the Warsaw University of Technology and the University of Warsaw.

The main aim of Euroanalysis XVII was to share ideas on recent developments of instrumentation and application in the field of analytical chemistry towards human well-being and sustainable development. Continuing previous successful conferences, both fundamental research and practical analytical applications were discussed.

The conference was held in Warsaw, a city of opportunities, entertainments, art, and science.

The conference began with well-attended short courses, organized for Sunday, 25th August. The opening ceremony conducted at the Great Hall at the Technical University of Warsaw was followed by the special plenary lecture given by Professor Adam Hulanicki, distinguished and worldwide recognized Polish analytical chemist. You can read his view on the history of Polish analytical chemistry in this issue.

A special session was dedicated to the DAC EuCheMS Award Ceremony of the Robert Kellner Lecture; this time the award was given to Jurgen Popp. After the special laudation given by Paul Worsfold, Raman spectroscopy and its use for biomedical analysis were intensely discussed by Jurgen Popp.

The conference program was divided into various thematic sessions, covering environmental, biomedical, forensic, and food analysis; trace elements and speciation; industrial and process analysis; separation techniques; electrochemical methods and devises; as well as recent progress in modern analysis. Analytical methods for cultural heritage and art, education in analytical chemistry as well as chemometrics, quality assurance, and metrology were discussed during the conference. The scientific program was concluded by the special keynote lecture on “Quantification of human errors in chemical analysis using expert judgments” given by I. Kuselman. Close to 600 posters were presented during the entire conference, and six of them were awarded Best Poster Awards by Springer. Besides the presentation given by senior scientists, it is worth highlighting the large number of young scientists and the high scientific level of their contributions.

The social program aimed to provide an occasion for informal discussion and enriching friendships with analytical chemistry societies. The key points were as follows: Sunday evening get-together in the main building of the Warsaw University of Technology to greet friends and welcome them to Warsaw; Beer Barbecue party in “Gościniec Wiecha” in Heritage Park outside of Warsaw, an excellent opportunity to taste local specialties of the Mazovian village cuisine on Monday evening; beautiful costumes, live folk music, and dance given by dancers on the stage of the famous “Teatr Polski” gave the chance for a music-and-dance trip around various regions of Poland during the enjoyable Tuesday evening; last but not least was the conference Gala Dinner, which took place on Wednesday in the Main Hall of the Warsaw University of Technology, which specially for this evening was turned into a unique ballroom complemented by a spectacular composition of light and music.

The very intense program of Euroanalysis made the days in Warsaw pass extremely quickly. During the closing ceremony, held on Friday, the participants expressed their appreciation to the organizing committee for all their effort to assure an excellent atmosphere during the conference.

The next Euroanalysis event will be organized in 2015, in Bordeaux; good luck to our friends from France!

